# Obstetrics and Gynecology

**Published:** 1895-03

**Authors:** 


### OBSTETRICS AND GYNECOLOGY.

EDITED BY DR. VIRGIL O. HARDON.

### REMOVAL OF THE UTERUS AND ADNEXA FOR PUERPERAL SEPSIS.

In 1887 the author had done abdominal section, removing the adherent ovaries and distended pus-tubes in a puerperal woman suffering from sepsis. The patient made a prompt recovery. This is the first case of this kind of which he had knowledge. He advocated that, where the ovary or tube is found distended with pus in the puerperal woman, the distended organ should at once be removed.

In the large class of cases where infection of the fallopian tube and ovary has occurred, and possibly of the peritoneum without the formation of pus, the treatment is to be determined, by two conditions: 1, the general condition of the patient; and 2, the ability of the physician to determine whether or not suppuration has occurred. In general it is safe to say that in any attack of puerperal salpingitis and puerperal peritonitis, no pus being present, immediate operation is not demanded. In those cases in which it is doubtful whether or not pus is present, and the general condition permitting, he would prefer to delay, watching the patient and operating later on if necessary.

There is another class of cases in which the patient suffers from puerperal fever without local signs of pelvic trouble. A certain number of these patients will inevitably die, unless the source of absorption is cut off. The only proper procedure in these cases is the removal of the uterus, thus stopping the absorption of sepsis, and unless sufficient has already been absorbed to disorganize the blood the patient stands a chance of recovery. The success will depend directly on the period of the disease at which the operation is performed. The earlier the operation, the greater the likelihood of a successful result. The author, however, believed that in the vast majority of septic cases seen in time, dangerous complications can be avoided by thorough curetting, irrigation, and antiseptic packing.

Conclusions: Patients suffering with puerperal septicemia, with pus in the appendages, should be submitted to abdominal section. If the disease is limited to one organ, only the affected organ should be removed. If it becomes necessary to remove both appendages, the general condition permitting, the uterus should be removed at the same time.

In cases of septicemia where suppuration has not taken place, the general condition not contraindicating, we should wait for the subsequent occurrence of symptoms.

Patients suffering from puerperal septicemia from the absorption of septic matters through the uterus whose lives are seriously threatened, will, in carefully selected cases, demand early abdominal section.—J. M. BALDY, M.D., in *Annals of Gynecology.*

---

### INFECTION OF THE URINARY TRACT AND BLADDER AFTER LABOR.

Infection of the urinary tract is not uncommon, and it may be of the ascending variety, beginning in the bladder or adjacent structures and extending to the ureter and kidneys; or of the descending variety, beginning primarily in the kidneys and passing to the ureter and bladder secondarily. The infecting poison may gain access in different ways, but usually by the catheter not being clean or by a clean catheter becoming contaminated by coming in contact with the lochial discharge. The healthy bladder can resist the action of bacteria, but in the puerperium this normal condition is not present on account of the contusions received by the bladder during labor. It has also been found that micro-organisms, in other of the pelvic viscera, may find their way into and infect the bladder. It is easy to understand how the kidneys may become infected by continuity of structure. The time required for the spread of infection from the bladder to the kidneys is usually ten days to two weeks.

The symptoms of septic puerperal cystitis are those of cystitis under other circumstances. The fever of cystitis generally passes over in a few days. Where the gradual depression of the temperature curve is followed by secondary rise accompanied by tenderness over the kidney, the diagnosis of secondary infection of the kidney is made out. In all cases of puerperal sepsis it is desirable that frequent examinations of the urine be made in order to detect the occurrence of ascending or descending nephritis, which may insidiously develop.

The prognosis of septic cystitis is favorable if proper treatment is begun early. The most important element in the treatment is the prevention. The use of the catheter should be delayed as long as possible. The danger of overdistention must also be borne in mind, for the traumatism resulting therefrom renders the bladder more susceptible to the influence of septic organisms. If the bladder is not evacuated within twelve hours, a clean catheter should be passed visually, with antiseptic cleansing. When cystitis develops, irrigation of the bladder should be employed at intervals of four hours, some mild antiseptic being employed, as creolin one-half per cent., or boric acid, fifteen grains to the ounce. Warm applications over the bladder and diluent drinks in connection with irrigation will usually check the cystitis in a few days.

With regard to infection of the rectum, the speaker said that the complication was a very rare one, but few cases being on record.—R. C. NORRIS, M.D., in *Annals of Gynecology.*

---

### THE CYSTITIS OF PREGNANCY.

The writer establishes in the first place the difference between cystitis of pregnant women and the so-called irritable bladder. The latter is above all characterized by a frequent and imperious desire to urinate. It appears either at the commencement or at the end of pregnancy. At the beginning of pregnancy the frequent desire to urinate is partly due to compression of the bladder, but especially to the congestion of the entire pelvis, which is very vascular during gestation, propagating itself to the bladder, and thus irritating it. At the end of gestation, the symptoms of irritable bladder are due to compression of the bladder by the uterus, and especially by the fetus. But the symptoms are not alarming and cease by rest in bed, a bland diet, and if necessary, a sitz bath. This is not the case in cystitis of pregnancy, and the characteristic points in this complication are frequent and most painful micturition. The desire to urinate is frequent and accompanied by pains, which radiate to the neighboring parts; the pains are increased by pressure over the hypogastric region, by fatigue, and cease or diminish by rest. The urine is changed in quality. By vaginal examination pain is produced when the ureter is pressed on. The urine, which is always normal in cases of irritable bladder, presents variable changes in the cystitis of pregnancy, thus distinguishing it from simple hemorrhagic or purulent cystitis. In the first instance, the urine only contains mucus and débris of vesical epithelium; in hemorrhagic cystitis, the urine is more or less red-colored, and contains red blood corpuscles; and lastly, if it is purulent, a whitish deposit is found at the bottom of the glass, and by the microscope pus cells are found. If the ammoniacal odor is very strong, it indicates a more serious condition. When purulent cystitis has become gangrenous, fragments of necrobic vesical mucous membrane may be found in the urine. As causes, compression of the bladder or ureters and pyelonephritis, with consecutive cystitis, have been invoked. But Prof. Tarnier believes that it is more likely due to retention of urine, so frequent during pregnancy, because retroversion of the gravid uterus can produce the same symptoms. The cystitis of pregnancy is only serious by its complications, which, for that matter, are incident to any kind of cystitis, and especially by abortion or premature labor that it may provoke. In delivered women, it sometimes has a fearful tenacity, and weakens the patient exceedingly, and if not following the cystitis it can only be attributed to the accoucheur, but a perfectly aseptic catheterism will not endanger the patient to this affection. Once developed, cystitis should be treated by the balsams, the following pills being often followed by an excellent result:

| | | |
|---|---|---|
| ℞ | Camphor .................................................................... | gr. xx. |
| | Opium.......................................................................... | gr. x. |

M. F. pil. No. xx.
S.—Take five or six pills daily.

If the cystitis is purulent, vesical antisepsis should be practiced by means of injections of a two per cent. boracic acid solution.—PROFESSOR TARNIER, in *Annals of Gynecology.*

### FIBROIDS AND CONCEPTION—PREGNANCY AND LABOR.

Dr. Hofmeier (*Zeitschr. f. Geburtsh. u. Gynäk*), in a very complete and well-tabulated essay, denies that fibroid disease of the uterus has any direct influence in causing sterility. Statistics do not show that, as has been alleged, subserous myoma predisposes considerably to sterility, whilst polypi and myoma of the cervix have little influence in that direction; nor can it be shown that fibroids promote fertility. These tumors seldom begin to appear till late in sexual life, so that if the patient is barren or a multipara the causes of her sterility or fecundity must have influenced her long before the development of her fibroid. The alleged frequency of this disease in elderly virgins is based on a fallacy. It is the local affection which the most readily drives a spinster to the gynecologist, whilst middle-aged married women trouble less about small and slow-growing abdominal swellings. Women with fibroids who marry late in sexual life are fairly fertile, though Hofmeier can hardly make out cause and effect in this fact. Fibroids by no means strongly predispose to abortion. It seems that this accident happens only when the uterine cavity is rendered unfit to bear through the size and relations of the tumor; nor does fibroid greatly interfere with the uterine contractions during labor. The best time for hysterectomy is not immediately after delivery, but a few weeks or months later. *St. Louis Medical and Surgical Journal.*

---

### CONDAMIN, R.: THE SURGICAL TREATMENT OF INOPERABLE CARCINOMA OF THE UTERUS (*Bull. du Lyon Méd.*, July 3, 1893).

The first portion of the paper deals with the limits within which operation is to be performed. Since every operator will decide upon each case according to his own experience and self-reliance, it would be idle to summarize. From the second part, which discusses the surgical treatment, stress need be laid only upon the advice to secure thorough dilatation before curetting for hemorrhages from the uterine cavity, and upon the author's preference for zinc chloride as a caustic. He claims to have observed that after thorough curetting, followed by a single cauterization with zinc chloride (1:2), in stick or paste, the carcinoma remained almost stationary for six months to a year and more. Antipyrin is strongly recommended for the pains.—I. F., in *American Journal Obstetrics.*

### LAPAROTOMY FOR OÖPHORITIS.

Dr. Asch, of Breslau (*Centralblatt fur Gynekology*), reports one hundred and seven cases of salpingo-ovaritis, in twenty-two of whom laparotomy was performed for removal of the ovary and tube of one or both sides. Of the twenty-two cases operated upon, two of the patients died; one from perforation of the intestine, the other from peritonitis occurring a month after the operation, the result of a gauze tampon accidentally left behind in the abdominal cavity. In the technique of operation for removal of the appendages in cases of pyosalpinx, Asch cauterizes the end of the fallopian tube, and then covers it with the peritoneum. Asch considers the French method of total extirpation of the uterus in diseases of the appendages an ideal operation in certain cases.—*St. Louis Med. and Surg. Journal.*

### PUERPERAL SEPSIS.

After a long and practical test, Professor E. P. Davis has concluded that the best method of combating the beginnings of puerperal sepsis is the following: After the first chill and rise in temperature a vaginal douche of hot water containing creolin (ʒi to Oi) or corrosive sublimate (1:4000) is given every four hours. At the end of twenty-four hours, if temperature has not reached normal, the interior of the uterus is curetted by means of the dull douche curette of Braun. By this means the uterine cavity is thoroughly flushed out with antiseptic fluid. Creolin (one dram to the pint of hot water) has generally been used. After the injection a suppository of 40 to 60 grains of iodoform in cocoa-butter has been inserted. In a few instances iodoform guaze has been used instead of the suppository.—*Polyclinic.*

